# Combining fecal immunochemical testing and questionnaire-based risk assessment in selecting participants for colonoscopy screening in the Chinese National Colorectal Cancer Screening Programs: A population-based cohort study

**DOI:** 10.1371/journal.pmed.1004340

**Published:** 2024-02-22

**Authors:** Xuesi Dong, Lingbin Du, Zilin Luo, Yongjie Xu, Chenran Wang, Fei Wang, Wei Cao, Liang Zhao, Yadi Zheng, Hongting Zhu, Changfa Xia, Jiang Li, Mulong Du, Dong Hang, Jiansong Ren, Jufang Shi, Hongbing Shen, Wanqing Chen, Ni Li, Jie He, Cancer Screening Program in Urban China (CanSPUC) Group

**Affiliations:** 1 Office of Cancer Screening, National Cancer Center/National Clinical Research Center for Cancer/Cancer Hospital, Chinese Academy of Medical Sciences and Peking Union Medical College, Beijing, China; 2 Chinese Academy of Medical Sciences Key Laboratory for National Cancer Big Data Analysis and Implement, Chinese Academy of Medical Sciences and Peking Union Medical College, Beijing, China; 3 Department of Cancer Prevention, Zhejiang Cancer Hospital, Hangzhou Institute of Medicine (HIM), Chinese Academy of Sciences, Hangzhou, Zhejiang, China; 4 Yongkang Center for Disease Control and Prevention, Yongkang, China; 5 Department of Epidemiology and Biostatistics, Jiangsu Key Lab of Cancer Biomarkers, Prevention and Treatment, Collaborative Innovation Center for Cancer Personalized Medicine, School of Public Health, Nanjing Medical University, Nanjing, China; Washington University in St Louis, UNITED STATES

## Abstract

**Background:**

Screening reduces colorectal cancer (CRC) burden by allowing early resection of precancerous and cancerous lesions. An adequate selection of high-risk individuals and a high uptake rate for colonoscopy screening are critical to identifying people more likely to benefit from screening and allocating healthcare resources properly. We evaluated whether combining a questionnaire-based interview for risk factors with fecal immunochemical test (FIT) outcomes for high-risk assessment is more efficient and economical than a questionnaire-based interview-only strategy.

**Methods and findings:**

In this multicenter, population-based, prospective cohort study, we enrolled community residents aged 40 to 74 years in 29 provinces across China. From 2016 to 2020, a total of 1,526,824 eligible participants were consecutively enrolled in the Cancer Screening Program in Urban China (CanSPUC) cohort, and 940,605 were enrolled in the Whole Life Cycle of Cancer Screening Program (WHOLE) cohort, with follow-up to December 31, 2022. The mean ages were 56.89 and 58.61 years in CanSPUC and WHOLE, respectively. In the WHOLE cohort, high-risk individuals were identified by combining questionnaire-based interviews to collect data on risk factors (demographics, diet history, family history of CRC, etc.) with FIT outcomes (RF–FIT strategy), whereas in the CanSPUC cohort, high-risk individuals were identified using only interview-based data on risk factors (RF strategy). The primary outcomes were participation rate and yield (detection rate of advanced neoplasm, early-stage detection rate of CRCs [stage I/II], screening yield per 10,000 invitees), which were reported for the entire population and for different gender and age groups. The secondary outcome was the cost per case detected.

In total, 71,967 (7.65%) and 281,985 (18.47%) individuals were identified as high-risk and were invited to undergo colonoscopy in the RF–FIT group and RF group, respectively. The colonoscopy participation rate in the RF–FIT group was 26.50% (19,071 of 71,967) and in the RF group was 19.54% (55,106 of 281,985; chi-squared test, *p* < 0.001). A total of 102 (0.53%) CRCs and 2,074 (10.88%) advanced adenomas were detected by the RF–FIT, versus 90 (0.16%) and 3,593 (6.52%) by the RF strategy (chi-squared test, both *p* < 0.001). The early-stage detection rate using the RF–FIT strategy was significantly higher than that by the RF strategy (67.05% versus 47.95%, Fisher’s exact test, *p* = 0.016). The cost per CRC detected was $24,849 by the RF–FIT strategy versus $55,846 by the RF strategy. A limitation of the study was lack of balance between groups with regard to family history of CRC (3.5% versus 0.7%).

**Conclusions:**

Colonoscopy participation and screening yield were better with the RF–FIT strategy. The association with CRC incidence and mortality reduction should be evaluated after long-term follow-up.

## Author summary

**Why was this study done?:**

➢ The burden of colorectal cancer (CRC) in China has been growing in recent years.
➢ More specific selection of individuals to undergo colonoscopy, based on both questionnaire-based risk assessment and fecal immunochemical test (FIT) results, could result in more efficiency allocation of colonoscopy resources.
➢ There is limited evidence on the colonoscopy screening yield when combining FITs and risk assessment through questionnaires.

**What did the researchers do and find?:**

➢ We calculated the baseline screening yield, participation, and cost per case detected in a national CRC screening program with a combined questionnaire-based risk assessment–FIT strategy.
➢ The baseline screening yield and participation rate were improved in the combined risk assessment–FIT group compared with risk factor assessment only group.
➢ The cost per CRC detected was $24,849 by the combined risk factor–FITs strategy, which was much lower than by the risk factor strategy ($55,846).

**What do these findings mean?:**

➢ A combined CRC screening strategy is feasible in China.
➢ The association between the combined RF-FIT strategy with CRC incidence and mortality reduction should be evaluated after long-term follow-up.

## Introduction

Colorectal cancer (CRC) is the third most common cancer worldwide [1]. In 2020, newly diagnosed CRC cases in China accounted for 28.8% of all new cases worldwide, and China accounted for 30.6% of all CRC-related deaths worldwide [1]. Screening reduces CRC burden, but there are widespread differences in CRC screening implementation strategies worldwide. Invitation to CRC screening based on age criteria (e.g., ≥50 years) is the mainstream practice, but this approach has been questioned in recent years [2]. The idea is that age alone should not guide invitation, but rather risk should be more comprehensively assessed [3].

Colonoscopy is considered by many to be the gold standard for CRC screening [4]. However, the low-value use of screening colonoscopy, such as colonoscopy overuse in average-risk populations, is wasteful and potentially harmful [5]. A recent randomized controlled trial (RCT) indicated that compared with a nonscreening group, colonoscopy screening among the average-risk population did not show superiority in CRC mortality reduction [6]. High-CRC-risk individuals selection, based on CRC risk assessment, could improve colonoscopy efficiency [3].

In 2012, the National Cancer Center of China (NCC) initiated the Cancer Screening Program in Urban China (CanSPUC), which identified the high-CRC-risk population by questionnaire-based interview for risk factors (RFs). We evaluated the screening yield from 2012 to 2015, but further improvements are needed in the participation rate and screening yield [7]. There is evidence that combining questionnaire-based interview and fecal immunochemical test (FIT) outcomes (RF–FIT) for high-risk individual identification could improve colonoscopy efficiency [3,8,9]. In 2019, RF–FIT strategy was explored in the Whole Life Cycle of Cancer Screening Program (WHOLE) [10]. In this study, we compared the exploratory RF–FIT strategy to the previous RF strategy to assess whether the yield and cost benefit of CRC screening program increased.

## Methods

### Study design

CanSPUC was initiated by the NCC in 2012 to target the 5 types of cancer most prevalent in China: lung cancer, female breast cancer, liver cancer, upper digestive tract cancer (esophageal cancer and gastric cancer), and CRC [7]. All data were transmitted to the coordinating center at NCC through a web-based management system belonging to the National Cancer Prevention and Control Network (NCPCN). Details about the data quality control are described in **S1 Text**.

The WHOLE was a nonprofit program consisting of several subprograms initiated by the NCC and provincial government in 2019. It targeted the same 5 types of cancer as CanSPUC but with a modified study protocol based on the subprogram’s study design [10]. Specifically, the FIT test was included in the risk assessment for CRC screening. All data from national and provincial nonprofit cancer screening programs were constantly transmitted to the coordinating center at NCC through NCPCN. Three provinces that did not perform FIT tests were not included in this study. The inclusion and exclusion criteria were in line with CanSPUC.

This study was approved by the ethics committees of China National Cancer Center/Cancer Hospital, Chinese Academy of Medical Sciences and Peking Union Medical College, and the ethics committees of each hospital from which participants were drawn (Number of IRB: 15-070/997 and 20/173-2369).

### Population recruitment

For both CanSPUC (RF strategy) and WHOLE (RF–FIT strategy), residents aged 40 to 74 years old were approached by trained staff by means of phone calls and personal encounters. Social media and community advertisements were used to raise public awareness of this cancer screening program. The study population was identified by the household registration system where all inhabitants of the regions are registered. All inhabitants had the same probability of being contacted by the staff. Residents who proactively contacted the staff to participate in the screening program were also included in the program to enhance public welfare. Residents without the following characteristics were encouraged to participate in CRC risk assessment: (a) weight loss of 10 pounds or more, or 5% of body weight, over a period of 6 to 12 months; (b) diagnosis of cancer; (c) history of colonic resection; (d) receipt of any cancer-related therapy (except for nonmelanoma skin cancer); (e) prior CRC screening; and (f) medical disability.

Staff members from the participating provinces distributed the informed consent and questionnaires among the participants, who were asked to complete the forms. After obtaining signed written informed consent, all eligible participants were interviewed to collect information about their exposure to risk factors including (1) demographics, (2) diet history, (3) lifestyle factors, (4) psychologic factors, and (5) medical history. In this study, we excluded incomplete questionnaires in which participants failed to provide an answer to two or more risk factor questions. The population enrolled during 2016 to 2018 (RF group) demonstrated a lower missing data rate than that during 2019 to 2020 (RF–FIT group). The main reason is that individuals in the RF group were recontacted by our staff to trace the missing data, which was not done in the RF–FIT group.

### Colorectal cancer risk assessment

Risk assessment based on questionnaire interview was performed in both RF and RF–FIT strategies, with follow-up to the 31st of December 2022. All the RFs utilized in this study were derived from large-scale national population evidence and subsequently subjected to expert review at NCC of China.

Three types of RFs were used in risk assessment: (1) demographics (age, gender [male or female], body mass index [BMI, >27 or ≤27], family history of CRC in first-degree relatives [Yes or No]); (2) diet history (dietary intake of whole grains [<2 kg/Month or ≥2 kg/Month], dietary intake of fresh vegetables [<10 kg/Month or ≥10 kg/Month], dietary intake of processed meat [<1.4 kg/Month or ≥1.4 kg/Month], habit of high-fat diet [Yes or No]); and (3) medical history (history of gallstones (Yes or No), history of chronic colitis [Yes or No], history of fecal occult blood test [Yes or No], and history of colonic polyps [Yes or No]). The details of risk score calculation was described in **S2 Text and S1 Table**. The cancer risk score system, which basically following the Harvard risk index (HRI), was utilized to assess an individual’s CRC risk through a questionnaire [11]. In the RF strategy, individuals with high risk scores over 1.5 were defined as having a high risk for CRC and were offered free colonoscopy. Low-risk individuals were not offered colonoscopy.

**Fig 1:**
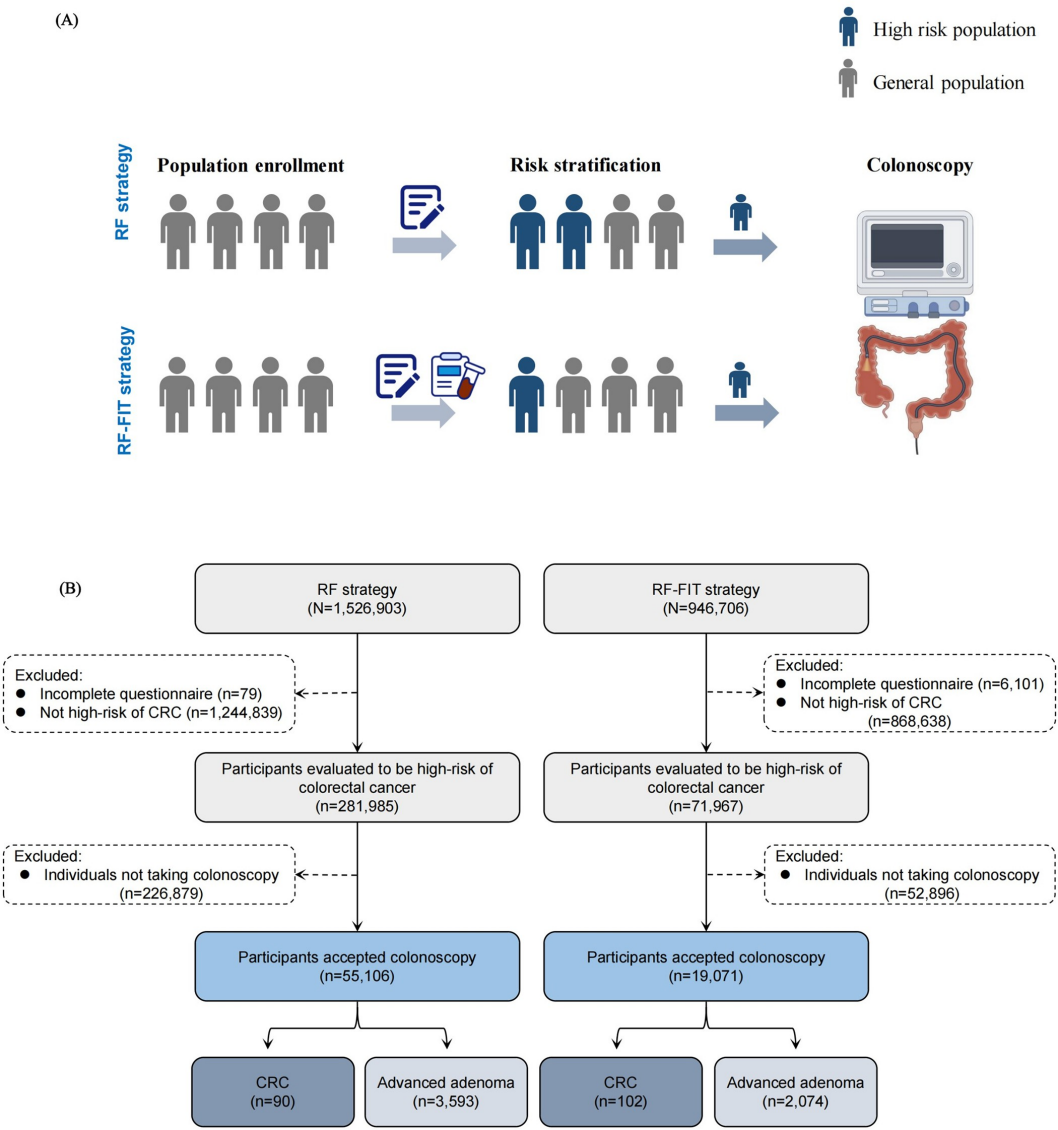
Study diagram and flow chart. (**A**) Diagram of study design. (**B**) Study flow chart. CRC, colorectal cancer; RF, risk factor; FIT, fecal immunochemical testing.

To enhance the efficiency of the questionnaire survey and mitigate potential recall bias in collecting diet history, we streamlined the cancer risk score system, preserving only demographic variables and medical history, and incorporated the FIT test into the RF–FIT strategy (**S2 Table**). In this study, only the series risk assessment approach was considered. Individuals with high risk scores and positive FIT results (cutoff = 100 ng/mL) in the RF–FIT strategy were defined as having a high risk for CRC. The FIT enabled visual interpretation of the test results as positive/negative by eye if the fecal hemoglobin concentration exceeded the threshold. The FIT test results were interpreted by trained staff, who then informed participants of the results. The study flow chart is displayed in **Fig 1A**.

### Colonoscopy

Colonoscopies were performed in a tertiary-level hospital by experienced gastroenterologists with the rank of attending physician or above and with at least 5 years of experience in colonoscopy. Biopsies were collected for further pathologic diagnosis. The Paris classification was used in the morphological examination.

### Primary and secondary outcomes

Participation rate and screening yield (detection rate of advanced neoplasm, early-stage detection rate of CRCs, screening yield per 10,000 invitees) were the primary outcomes of interest in this study. Advanced neoplasms included CRC and advanced adenomas. The International Classification of Diseases 10th Revision codes were used throughout, in which CRC was coded as C18-C21. Early-stage CRCs in this study were stage I/II CRCs. Advanced adenomas included (1) at least one adenoma ≥10 mm, (2) one with villous components, or (3) high-grade dysplasia. Pathological examination after colonoscopy is described in **S3 Text**. The cost per case detected in screening was a secondary outcome of interest in this study.

### Statistical analysis

Baseline characteristics were assessed using standardized mean differences (SMDs). SMD>0.2 was considered a significant difference. The *p* values were calculated by *t* test or Wilcoxon rank-sum test for continuous variables and the chi-squared test or Fisher’s exact test for categorical variables. The screening yield, including the detection rate of advanced neoplasm (1), early-stage detection rate of CRCs (2), screening yield per 10,000 invitees (3), and colonoscopies to detect one lesion (4), was calculated as follows.


$$Detection\;rate\;of\;advanced\;neoplasm=\frac{Number\;of\;people\;with\;advanced\;neoplasm}{Number\;of\;people\;received\;colonoscopy\;screening}$$ (1)

$$Detection\;rate\;of\;early\;stage\;CRCs=\frac{Number\;of\;stage\;I\;or\;II\;CRCs}{Number\;of\;CRCs}$$ (2)

Yieldper10,000invitees=10,000×Participationrate×Detectionrateofadvancedneoplasm (3)

$$Colonoscopies\;to\;detect\;one\;lesion=\frac{1}{Detection\;rate\;of\;advanced\;neoplasm}$$ (4)

Cost analysis was conducted from the government’s perspective, considering that CanSPUC is a single-payer screening program. The government pays $0.68 per completed risk assessment, $2.17 per FIT, and $72.47 per colonoscopy. All costs were collected in the Chinese yuan and converted to US dollars for this publication (CNY = 6.8996 per US$1, the yearly average currency exchange rate of 2020). Statistical analyses were performed with R V.4.1.2. Missing data were not imputed, which was different from our previous study [10]. All tests were two-tailed, and *P* values ≤ 0.05 were considered statistically significant. This study is reported as per the Strengthening the Reporting of Observational Studies in Epidemiology (STROBE) guidelines **(S3 Table)**.

## Results

1,526,903 and 946,706 participants were enrolled in the RF strategy and RF–FIT strategy, respectively. After excluding participants with incomplete risk assessments, 1,526,824 participants were included in the RF strategy and 940,605 participants were included in the RF–FIT strategy. The characteristics of the enrolled populations are described in **Table 1**. The baseline characteristics were comparable (SMD<0.2) between the RF strategy and the RF–FIT strategy. The study flowchart is displayed in **Fig 1B**. 281,985 (18.47%) of 1,526,824 individuals assessed using the RF strategy and 71,967 (7.65%) of 940,605 individuals assessed using the RF-FIT strategy were identified as having high-CRC-risk and recommended for free colonoscopy.

**Table 1:** Participant characteristics.

Characteristics	Level	Overall	RF strategy	RF–FIT strategy	*p* value	SMD
		*N* = 2,467,429	*N* = 1,526,824	*N* = 940,605		
Age, mean (SD)		57.55 (8.96)	56.89 (9.43)	58.61 (8.03)	<0.001	0.19
Gender (%)	Female	1,451,716 (58.8)	890,129 (58.3)	561,587 (59.7)	<0.001	0.03
	Male	1,015,713 (41.2)	636,695 (41.7)	379,018 (40.3)		
BMI (%)	Normal	1,573,862 (64.1)	980,780 (64.6)	593,082 (63.3)	<0.001	0.03
	Obesity	96,736 (3.9)	57,058 (3.8)	39,678 (4.2)		
	Overweight	757,272 (30.8)	464,507 (30.6)	292,765 (31.3)		
	Underweight	27,096 (1.1)	16,012 (1.1)	11,084 (1.2)		
Smoking status (%)	Current	375,426 (15.2)	215,698 (14.1)	159,728 (17.0)	<0.001	0.09
	Ever	97,031 (3.9)	55,170 (3.6)	41,861 (4.5)		
	Never	1,994,966 (80.9)	1,255,956 (82.3)	739,010 (78.6)		
Alcohol intake (%)	Current/Ever	400,243 (16.2)	273,363 (17.9)	126,880 (13.5)	<0.001	0.12
	Never	2,067,157 (83.8)	1,253,460 (82.1)	813,697 (86.5)		
Physical exercise (%)	No	1,440,354 (58.4)	942,816 (61.8)	497,538 (52.9)	<0.001	0.18
	Yes	1,027,058 (41.6)	584,008 (38.2)	443,050 (47.1)		
Family history for CRC (%)	No	2,405,500 (97.5)	1,473,058 (96.5)	932,442 (99.3)	<0.001	0.19
	Yes	60,736 (2.5)	53,766 (3.5)	6,970 (0.7)		
Intestinal disease (%)	No	2,196,878 (89.1)	1,351,621 (88.5)	845,257 (90.0)	<0.001	0.05
	Yes	269,057 (10.9)	175,185 (11.5)	93,872 (10.0)		

BMI, body mass index (normal: 18–24.9, overweight: 25–29.9, obesity: ≥30, underweight: <18); CRC, colorectal cancer; FIT, fecal immunochemical test; RF, risk factor; SMD, standardized mean difference. The missing data in CanSPUC (RF strategy) was not imputed, which was different from our previous study [10]. In RF strategy, data were missing for BMI for 8,467 participants, alcohol intake for 1, and intestinal disease for 18. In RF–FIT strategy, data were missing for BMI for 3,996 participants, smoking status for 6, alcohol intake for 28, physical exercise for 17, family history for CRC for 1,193, and intestinal disease for 1,476.

High-CRC-risk individuals in the RF–FIT strategy group tended to be older than those in the RF strategy group (58.81 [7.81] versus 55.46 [8.65], SMD = 0.41; Table 2). Compared with the RF strategy, the RF–FIT strategy identified more high-CRC-risk individuals with less exposure to risk factors. Specifically, high-CRC-risk individuals identified using the RF–FIT approach showed less first-degree family history of CRC (0.9% versus 4.8%, SMD = 0.24), less alcohol intake (23.8% versus 45.2%, SMD = 0.46), less intestinal disease (30.6% versus 58.3%, SMD = 0.58), and more physical exercise than high-risk individuals identified using the RF strategy (46.1% versus 30.3%, SMD = 0.33).

**Table 2:** Characteristics of high colorectal cancer risk participants identified using the risk factor (RF) or RF–fecal immunochemical test (FIT) strategies.

Characteristics	Level	Overall	RF strategy	RF–FIT strategy	*p* value	SMD
		(*N* = 353,952)	(*N* = 281,985)	(*N* = 71,967)		
Age, mean (SD)		56.14 (8.59)	55.46 (8.65)	58.81 (7.81)	<0.001	0.41
Gender (%)	Female	207,134 (58.5)	165,836 (58.8)	41,298 (57.4)	<0.001	0.03
	Male	146,818 (41.5)	116,149 (41.2)	30,669 (42.6)		
BMI (%)	Normal	202,800 (57.3)	160,007 (56.7)	42,793 (59.5)	<0.001	0.08
	Obesity	20,184 (5.7)	16,753 (5.9)	3,431 (4.8)		
	Overweight	124,732 (35.2)	100,076 (35.5)	24,656 (34.3)		
	Underweight	3,849 (1.1)	3,042 (1.1)	807 (1.1)		
Smoking status (%)	Current	102,448 (28.9)	84,756 (30.1)	17,692 (24.6)	<0.001	0.12
	Ever	19,296 (5.5)	14,926 (5.3)	4,370 (6.1)		
	Never	232,208 (65.6)	182,303 (64.6)	49,905 (69.3)		
Alcohol intake (%)	Current/Ever	144,575 (40.8)	127,415 (45.2)	17,160 (23.8)	<0.001	0.46
	Never	209,377 (59.2)	154,570 (54.8)	54,807 (76.2)		
Physical exercise (%)	No	235,324 (66.5)	196,549 (69.7)	38,775 (53.9)	<0.001	0.33
	Yes	118,628 (33.5)	85,436 (30.3)	33,192 (46.1)		
Family history of CRC (%)	No	339,839 (96.0)	268,494 (95.2)	71,345 (99.1)	<0.001	0.24
	Yes	14,113 (4.0)	13,491 (4.8)	622 (0.9)		
Intestinal disease (%)	No	167,549 (47.3)	117,627 (41.7)	49,922 (69.4)	<0.001	0.58
	Yes	186,391 (52.7)	164,346 (58.3)	22,045 (30.6)		

BMI, body mass index (normal: 18–24.9, overweight: 25–29.9, obesity: ≥30, underweight: <18); CRC, colorectal cancer; FIT, fecal immunochemical testing; RF, risk factor; SMD, standardized mean difference. In RF strategy, data were missing for BMI for 2,107 participants and intestinal disease for 12. In RF–FIT strategy, data were missing for BMI for 280 participants.

Among individuals identified as having high-CRC risk, 55,106 (19.54%) individuals in the RF strategy group and 19,071 (26.50%) in the RF-FIT strategy group adhered to colonoscopy (*p* < 0.001). Rates of adherence to colonoscope, stratified by risk factor groups is shown in **Table 3**. Colonoscopy uptake was significant higher in the RF-FIT strategy group across the age groups (6.93% to 14.22%, *p* < 0.001). For participants with other characteristics (e.g., gender, BMI, smoking status, alcohol intake, physical exercise, intestinal disease), participation rates were also higher in the RF-FIT strategy group, ranging from 4.34% to 14.69%. However, the participation rate for individuals with a family history of CRC did not show a significant difference between the two groups.

**Table 3:** Colonoscopy screening participation rate in risk factor (RF) and RF–fecal immunochemical test (FIT) strategies.

Character	Level	RF strategy		RF–FIT strategy		Improvement (%)	*p* value
		High risk for CRC	Participants undertaking colonoscopy (%)	High risk for CRC	Participants undertaking colonoscopy (%)		
**Population**		281,985	55,106 (19.54)	71,967	19,071 (26.50)	**6.96**	<0.001
**Age**	40–44	34,269	6,657 (19.43)	324	109 (33.64)	**14.22**	<0.001
	45–49	47,408	10,210 (21.54)	10,293	3,032 (29.46)	**7.92**	<0.001
	50–54	54,348	11,947 (21.98)	13,119	3,963 (30.21)	**8.23**	<0.001
	55–59	44,420	9,410 (21.18)	14,226	4,111 (28.90)	**7.71**	<0.001
	60–64	50,352	9,528 (18.92)	13,231	3,420 (25.85)	**6.93**	<0.001
	65–69	37,111	5,712 (15.39)	13,963	3,150 (22.56)	**7.17**	<0.001
	70–74	14,077	1,642 (11.66)	6,811	1,286 (18.88)	**7.22**	<0.001
**Gender**	Female	165,836	32,593 (19.65)	41,298	10,857 (26.29)	**6.64**	<0.001
	Male	116,149	22,513 (19.38)	30,669	8,214 (26.78)	**7.40**	<0.001
**BMI**	Normal	160,007	32,418 (20.26)	42,793	11,572 (27.04)	**6.78**	<0.001
	Obesity	16,753	2,641 (15.76)	3,431	763 (22.24)	**6.47**	<0.001
	Overweight	100,076	19,123 (19.11)	24,656	6,478 (26.27)	**7.17**	<0.001
	Underweight	3,042	569 (18.70)	807	186 (23.05)	**4.34**	<0.001
**Smoking status**	Current	84,756	16,389 (19.34)	17,692	4,964 (28.06)	**8.72**	<0.001
	Ever	14,926	3,073 (20.59)	4,370	1,152 (26.36)	**5.77**	<0.001
	Never	182,303	35,644 (19.55)	49,905	12,955 (25.96)	**6.41**	<0.001
**Alcohol intake**	Current/ever	127,415	26,258 (20.61)	17,160	4,828 (28.14)	**7.53**	<0.001
	Never	154,570	28,848 (18.66)	54,807	14,243 (25.99)	**7.32**	<0.001
**Physical exercise**	No	196,549	40,263 (20.48)	38,775	10,225 (26.37)	**5.89**	<0.001
	Yes	85,436	14,843 (17.37)	33,192	8,846 (26.65)	**9.28**	<0.001
**Intestinal disease**	No	117,627	17,672 (15.02)	49,922	10,811 (21.66)	**6.63**	<0.001
	Yes	164,346	37,429 (22.77)	22,045	8,260 (37.47)	**14.69**	<0.001
**Family history of CRC**	No	268,494	51,280 (19.10)	71,345	18,892 (26.48)	**7.38**	<0.001
	Yes	13,491	3,826 (28.36)	622	179 (28.78)	0.42	0.857

BMI, body mass index (normal: 18–24.9, overweight: 25–29.9, obesity: ≥30, underweight: <18); CRC, colorectal cancer; FIT, fecal immunochemical test; RF, risk factor. Chi-squared test was used to calculate the *p* values.

**Fig 2:**
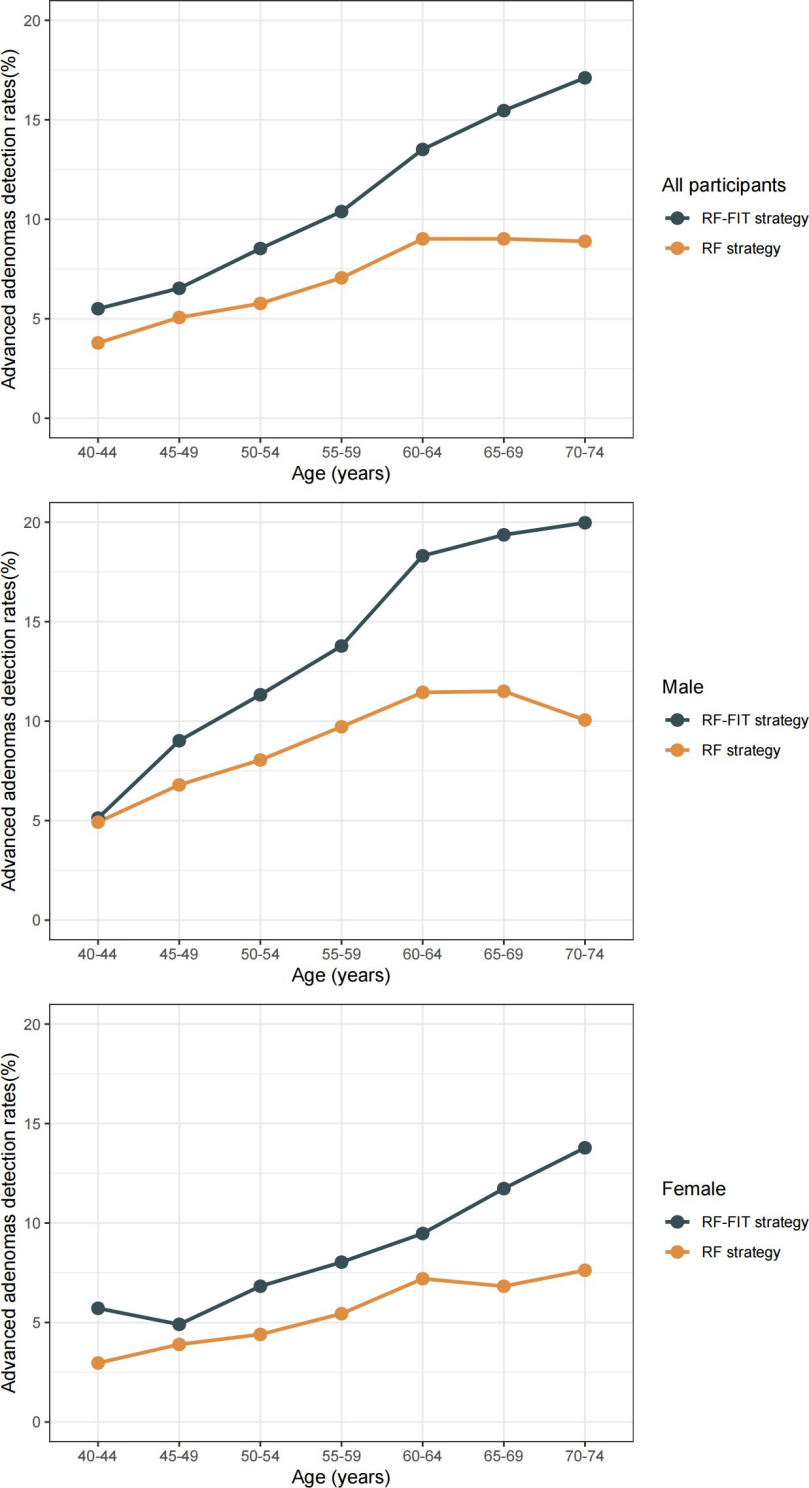
Advanced neoplasms detection rates in risk factor (RF) and RF-fecal immunochemical test (FIT) strategies.

**Fig 3:**
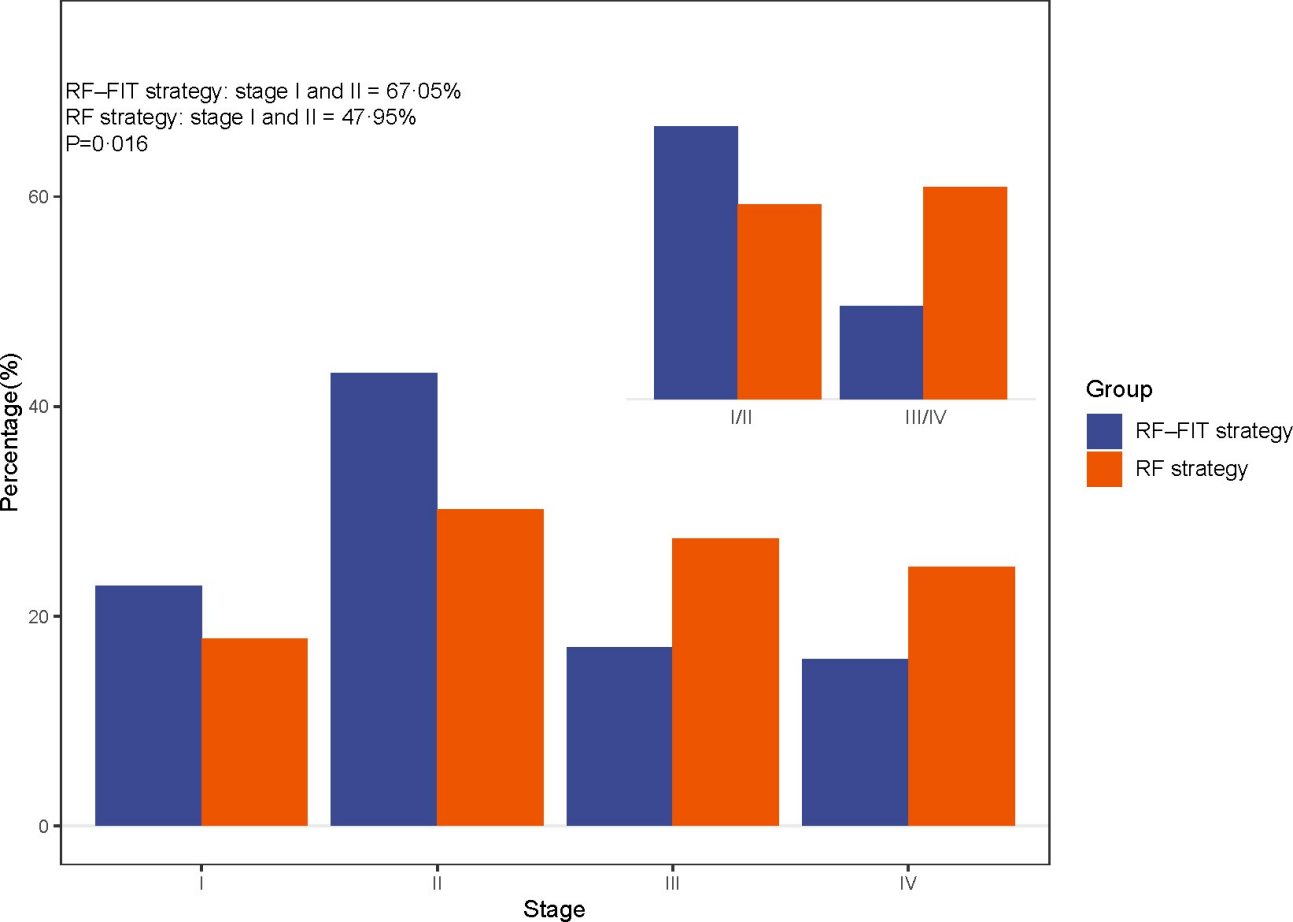
The percentage of early-stage colorectal cancer detection in risk factor (RF) and RF-fecal immunochemical test (FIT) strategies.

Advanced neoplasm detection rates in the RF–FIT group were significantly higher than those in the RF group (Table 4). Specifically, 102 (0.53%) CRCs and 2,074 (10.88%) advanced adenomas were detected in the RF–FIT group, which was significantly higher than in the RF group (CRCs: 90 [0.16%]; advanced adenomas: 3,593 [6.52%], both *p* < 0.001). Detection rates for advanced neoplasms increased with age in both groups (Fig 2). The overall trend for detection rates in the RF–FIT group was higher than that in the RF group, with similar trends in both men and women (*p* < 0.001). The detection rate of early-stage CRC is presented in **Fig 3**. The percentage of stage I-II CRC in the RF–FIT group was much higher than that in the RF group (67.05% [59/88; 14 CRCs were missing the stage], versus 47.95% [35/73; 17 CRCs were missing the stage], *p* = 0.016).

**Table 4:** Screening yield of colonoscopy screening in risk factor (RF) and RF–fecal immunochemical test (FIT) strategies.

Finding	RF strategy (*n* = 55,106)				RF–FIT strategy (*n* = 19,071)			*p* value**
	Detected cases (%)	Yield per 10,000 invitees	Colonoscopies to detect one lesion (n)	Detected cases (%)		Yield per 10,000 invitees	Colonoscopies to detect one lesion (n)	
CRC	90(0.16)	4	625	102 (0.53)		15	189	<0.001
Advanced adenoma*	3,593(6.52)	127	16	2,074 (10.88)		289	10	<0.001
at least one adenoma ≥10 mm	1,571(2.85)	56	36	1,724 (9.04)		240	12	<0.001
at least one adenoma with villous components	419(0.76)	15	132	298 (1.56)		42	65	<0.001
at least one adenoma with high-grade dysplasia	1,743(3.16)	62	32	87 (0.46)		13	218	<0.001
Other benign lesions	5,247 (9.52%)	187	11	1,896 (9.94)		263	11	0.093

The invitees refer to the high-CRC-risk population who are invited to receive colonoscopy.

* Various subtypes of advanced adenomas could coexist in the same individual, thus the total number of individuals with advanced adenomas does not equal the total number of each subtype.

**P value represents the difference of detected cases between RF strategy and RF–FIT strategy.

Chi-squared test was used to calculate the *p* value.

CRC, colorectal cancer; FIT, fecal immunochemical test; RF, risk factor.

As a result, 191 and 625 colonoscopies were needed to detect one CRC by the RF–FIT strategy and RF strategy, respectively, and 10 and 16 colonoscopies were needed to detect one advanced adenoma by the RF–FIT strategy and RF strategy, respectively. Furthermore, the screening yield per 10,000 invitees by the RF–FIT strategy was superior to that of the RF strategy, both for CRC (15 versus 4) and for advanced adenoma (289 versus 127). The detection rate, colonoscopies to detect one lesion, and screening yield of benign lesions for the RF–FIT strategy were superior to those for the RF strategy.

The overall study cost per participant, per advanced neoplasm detected, per CRC case detected, and per advanced adenoma detected by the RF–FIT strategy were $2.69, $1,164.80, $24,849.00, and $1,222.08, respectively, which were 18.14%, 14.65%, 55.50%, and 12.64% lower than those of the RF strategy (**Table 5**).

**Table 5:** Cost analysis from a government perspective in risk factor (RF) and RF–fecal immunochemical test (FIT) strategies.

Term	RF strategy	RF–FIT strategy	Percentage reduction (%)
Participants provided with qualified risk assessment	1,526,824	940,605	-
Participants provided with FIT	-	237,515	-
Participants undertaking colonoscopy	55,106	19,071	-
Overall cost (US$)	5,026,114	2,534,598	-
Cost per participant (US$)	3.29	2.69	18.14
Cost per advanced neoplasia detected (US$)	1,364.68	1,164.80	14.65
Cost per CRC case detected (US$)	55,845.72	24,849.00	55.50
Cost per advanced adenoma detected (US$)	1,398.86	1,222.08	12.64

CRC, colorectal cancer; FIT, fecal immunochemical test; RF, risk factor.

## Discussion

In this study, we proposed and evaluated a CRC screening strategy from a national screening program in the baseline phase. The RF–FIT strategy identified people with high CRC risk who should thus undergo colonoscopy based on both CRC RFs and FIT outcome, and this approach demonstrated a higher colonscopy participation rate and screening yield compared with the RF only approach and was cost-effective. The cost per CRC detected was $24,849 by the RF–FIT strategy, which was much lower than that by the RF strategy ($55,846).

A recent study measured the overuse of colonoscopy in the United States Department of Veterans Affairs health system, which provided colonoscopies as first-line screening to people at average risk of CRC [5]. Surprisingly, more than 24% of colonoscopies were suspected to be overused, which was in line with the results from a recent systematic review of studies measuring overuse of colonoscopy in the USA [12]. The lack of high-quality evidence on the benefit of colonoscopy for first-line screening has led to Australian and Canadian guidelines advising against it [13]. Therefore, we wanted to develop a risk-adapted strategy for high-CRC-risk individuals preselection before colonoscopy. In theory, the preselection of high-CRC-risk populations would reduce the proportion of colonoscopy that are overused. In fact, a risk-adapted strategy is recommended in most situations to conserve resources and avoid colonoscopy overuse, especially in locales with limited health resources [3,9,14].

Several studies have reported the usefullness of FIT-based risk-adapted strategies in CRC screening. However, to our knowledge, there are limited systematic evaluations in national screening programs. Such evaluations could offer complementary evidence to that of RCTs in practical settings [15]. Indeed, RCTs constitute the highest level of evidence to inform guideline development for CRC screening, but evidence from screening practices and RCTs is considered mutually complementary. Not all research questions regarding CRC screening can be addressed through RCTs [16]. For example, researchers cannot intervene to identify disparities in access to CRC screening and identify the real gains in screening practice.

The Asia Pacific Working Group recommended a preliminary risk assessment to identify high-risk populations before colonoscopy [17]. The current mainstream preliminary risk assessment tools include questionnaire-based risk assessment and fecal occult blood tests (e.g., FIT, guaiac fecal occult blood test (gFOBT)) [17]. As a recent study demonstrated, a questionnaire-based risk assessment combined with a fecal hemoglobin test increased the ensuing colonoscopy participation rate by 6.84% [18]. In this study, a significant improvement (6.94%) in the participation rate was also observed with the combined RF–FIT strategy. Depending on the arrangement, high-CRC-risk individuals should be informed of the risk assessment results and the benefits and potential harms of colonoscopy screening, and recommended to undergo colonoscopy as appropriate. The increased colonoscopy uptake rate among individuals in the RF–FIT strategy group could be due to several reasons. First, the staff can explain the results of FIT tests to participants in a more straightforward manner, thereby decreasing the difficulty of communication and mobilization for screening. Second, when community staff discuss the FIT results with residents, the residents can intuitively sense the importance of undergoing CRC screening.

A meta-analysis that evaluated 17 original CRC risk scores separately reported limited AUCs (0.62 to 0.77) in advanced colorectal neoplasia prediction [19]. The ability of CRC risk scores to identify high-risk populations might have been better. Another study reported that FIT could be a critical supplement to the risk scores [20]. One study showed that a questionnaire and FIT had a higher yield than the questionnaire alone, with better identification of CRC high-risk populations. The yield of advanced neoplasms per 10,000 invitees was 46.9 and 12.2, respectively, and the number of colonoscopies needed to detect one advanced neoplasm was 11.4 and 28.4, respectively [21].

The cost paid for by the government is an important consideration in nationally organized screening programs. Compared to the RF-only strategy, the combined strategy cost the government less. In a Nigerian study that treated FIT as the screening modality, the programmatic cost per advanced neoplasia and CRC case detected was $5,686 and $43,591, respectively [22], which is higher than the cost of the RF–FIT strategy in our study.

This investigation has several strengths. The major strength is that this study is the largest risk-adapted national CRC screening program worldwide, which could be considered an essential complement for RCTs. Our analyses may pave the way for more efficient CRC screening in nationwide population-based screening programs. Additionally, the screening program in this study is sourced from centers across China, giving this study high representativeness. Some provinces, such as Zhejiang, have been conducting CRC screenings since the last century. They have extensive operational experience and reliable data quality, providing technical references and data assurance for the implementation of our new strategy. Finally, RF–FIT strategy is the only exploratory practice in national CRC screening program of China. Based on the WHOLE program, we will offer a variety of methods to identified high-CRC-risk individuals and give more results of screening strategies.

This investigation has several limitations. The major limitation is that the cohorts were not randomized but were recruited sequentially from a fixed set of provinces, which is a potentially significant risk of bias. The national CRC screening programs have been ongoing in these fixed areas for over a decade, which means that individuals with critical risk factors (e.g., family history of CRC) may have been more likely to participate in the screening program during its early stages. Although the SMD of risk factors was not significant (lower than 0.2) between the groups, we still observed fewer participants with certain RFs (e.g., family history of CRC, alcohol intake) recruited in RF–FIT strategy (2019 to 2020). Another limitation of this study was the short follow-up time. Thus, the current study cannot evaluate CRC incidence or mortality reduction. However, the combined strategy could identify more early-stage CRCs, which is likely to results in reduced mortality. The gains in mortality and incidence reduction will be evaluated in the future. Additionally, considering the high demand for endoscopists and the regional disparities in medical technology within the national screening program, we did not establish strict nationwide parameters for endoscopists concerning intubation rates and adenoma detection rates. However, at the outset of inclusion, individual endoscopists in both groups were in full agreement, which helped mitigate potential bias. Finally, the population enrolled during 2016 to 2018 (RF group) demonstrated a lower missing data rate than that during 2019 to 2020 (RF–FIT group). The main reason is that individuals in RF group were recontacted by our staff to trace the missing data, which was not done in the RF–FIT group. In future studies, we will recontact the RF–FIT group and impute the missing data as much as possible.

This study reported a feasible strategy for CRC screening in China and other settings where risk assessments are being considered. Combining a questionnaire-based interview for RFs with FIT outcomes for high-risk assessment is more efficient and economical than relying solely on a questionnaire-based interview strategy. The association with CRC incidence and mortality reduction should be evaluated after long-term follow-up.

### Ethical statement

The study was approved by the Ethics Committee of National Cancer Center/Cancer Hospital, Chinese Academy of Medical Sciences, and Peking Union Medical College (IRB number: 15-070/997 and 20/173-2369), and all participants provided written informed consent.

## Supporting information

S1 Table The risk factor and its relative risk in CanSPUC (RF strategy). (DOCX)

S2 Table The risk factor and its risk score in WHOLE (RF–FIT strategy). (DOCX)

S3 Table STROBE Statement—Checklist of items that should be included in reports of cohort studies. (DOCX)

S1 Text Quality control. (DOCX)

S2 Text Colorectal cancer risk score calculation. (DOCX)

S3 Text Pathological examination. (DOCX)

## Abbreviations

BMI: body mass index
CanSPUC: Cancer Screening Program in Urban China
CRC: colorectal cancer
FIT: fecal immunochemical test
gFOBT: guaiac fecal occult blood test
HRI: Harvard risk index
NCC: National Cancer Center of China
NCPCN: National Cancer Prevention and Control Network
RCT: randomized controlled trial
RF: risk factor
SMD: standardized mean difference
WHOLE: Whole Life Cycle of Cancer Screening Program
