# Supplementary material for: Combining fecal immunochemical testing and questionnaire-based risk assessment in selecting participants for colonoscopy screening in the Chinese National Colorectal Cancer Screening Programs: A population-based cohort study
Source: PLoS Med. 2024 Feb 22;21(2):e1004340. doi: 10.1371/journal.pmed.1004340 (PMC10883529; doi:10.1371/journal.pmed.1004340)
Supplement: S1 Table — (DOCX) [file pmed.1004340.s001.docx]

**S1 Table. The risk factor and its relative risk in CanSPUC (RF strategy)**

| **Risk factors** | **Relative Risk** | | **Prevalence** | |
| --- | --- | --- | --- | --- |
|  | **Male** | **Female** | **Male** | **Female** |
| Body mass index（BMI） |  |  |  |  |
| *＞ 27* | 1.5 | 1.5 | 0.071 | 0.071 |
| Dietary intake of whole grains |  |  |  |  |
| *＜2kg/Month* | 1.538 | 1.538 | 0.91 | 0.905 |
| Dietary intake of fresh vegetables |  |  |  |  |
| *＜10kg/Month* | 1.328 | 1.328 | 0.516 | 0.527 |
| Dietary intake of processed meat |  |  |  |  |
| *＞1.4kg/Month* | 1.5 | 1.5 | 0.697 | 0.589 |
| Habit of high-fat diet |  |  |  |  |
| *Yes* | 2.17 | 2.17 | 0.3 | 0.2 |
| History of gallstones |  |  |  |  |
| *Yes* | 4.6 | 4.6 | 0.051 | 0.086 |
| History of chronic colitis |  |  |  |  |
| *Yes* | 13.656 | 13.656 | 0.016 | 0.016 |
| Family history of colorectal cancer in first-degree relatives |  |  |  |  |
| *Yes* | 2.44 | 2.44 | 0.0338 | 0.0338 |
