# Supplementary material for: Combining fecal immunochemical testing and questionnaire-based risk assessment in selecting participants for colonoscopy screening in the Chinese National Colorectal Cancer Screening Programs: A population-based cohort study
Source: PLoS Med. 2024 Feb 22;21(2):e1004340. doi: 10.1371/journal.pmed.1004340 (PMC10883529; doi:10.1371/journal.pmed.1004340)
Supplement: S2 Table — (DOCX) [file pmed.1004340.s002.docx]

**S2 Table. The risk factor and its risk score in WHOLE (RF-FIT strategy)**

| **Risk factors** | **Level** | **Score** |
| --- | --- | --- |
| Age,yrs | *≤54* | 0 |
|  | *55-64* | 1 |
|  | *≥65* | 2 |
| Gender | *Female* | 0 |
|  | *Male* | 1 |
| Family history of colorectal cancer in first-degree relatives | *No* | 0 |
|  | *Yes* | 1 |
| Smoking status | *Never* | 0 |
|  | *Current/ever* | 1 |
| Body mass index (BMI) | *<23* | 0 |
|  | *≥23* | 1 |
