# Supplementary material for: Combining fecal immunochemical testing and questionnaire-based risk assessment in selecting participants for colonoscopy screening in the Chinese National Colorectal Cancer Screening Programs: A population-based cohort study
Source: PLoS Med. 2024 Feb 22;21(2):e1004340. doi: 10.1371/journal.pmed.1004340 (PMC10883529; doi:10.1371/journal.pmed.1004340)
Supplement: S3 Table — (DOCX) [file pmed.1004340.s003.docx]

**S3 Table: STROBE Statement—Checklist of items that should be included in reports of cohort studies**

|  | Item No | Recommendation |
| --- | --- | --- |
| **Title and abstract** | 1 | (*a*) Indicate the study’s design with a commonly used term in the title or the abstract Title page and Abstract page (Methods and Findings) |
|  |  | (*b*) Provide in the abstract an informative and balanced summary of what was done and what was found  Abstract page (Methods and Findings) |
| Introduction | | |
| Background/rationale | 2 | Explain the scientific background and rationale for the investigation being reported Introduction (4^th^ paragraph) |
| Objectives | 3 | State specific objectives, including any prespecified hypotheses Introduction (5^th^ paragraph) |
| Methods | | |
| Study design | 4 | Present key elements of study design early in the paper Methods (1-2 paragraphs) |
| Setting | 5 | Describe the setting, locations, and relevant dates, including periods of recruitment, exposure, follow-up, and data collection Methods (1-2 paragraphs) and Supplementary materials (S1: Recruitment process) |
| Participants | 6 | (*a*) Give the eligibility criteria, and the sources and methods of selection of participants. Describe methods of follow-up Methods (1-2 paragraphs) |
|  |  | (*b*) For matched studies, give matching criteria and number of exposed and unexposed Not applicable |
| Variables | 7 | Clearly define all outcomes, exposures, predictors, potential confounders, and effect modifiers. Give diagnostic criteria, if applicable Methods (Primary and secondary outcomes, 1st paragraph) |
| Data sources/ measurement | 8* | For each variable of interest, give sources of data and details of methods of assessment (measurement). Describe comparability of assessment methods if there is more than one group Methods (Primary and secondary outcomes) |
| Bias | 9 | Describe any efforts to address potential sources of bias Supplementary materials (S3: Quality control) |
| Study size | 10 | Explain how the study size was arrived at Methods (1-2 paragraphs) |
| Quantitative variables | 11 | Explain how quantitative variables were handled in the analyses. If applicable, describe which groupings were chosen and why Methods (statistical analysis) |
| Statistical methods | 12 | (*a*) Describe all statistical methods, including those used to control for confounding Methods (statistical analysis) |
|  |  | (*b*) Describe any methods used to examine subgroups and interactions Methods (statistical analysis) |
|  |  | (*c*) Explain how missing data were addressed Supplementary materials (Table S2) |
|  |  | (*d*) If applicable, explain how loss to follow-up was addressed Not applicable |
|  |  | (*e*) Describe any sensitivity analyses Supplementary materials (Table S2) |
| Results | | |
| Participants | 13* | (a) Report numbers of individuals at each stage of study—eg numbers potentially eligible, examined for eligibility, confirmed eligible, included in the study, completing follow-up, and analysed Results (1st paragraph) |
|  |  | (b) Give reasons for non-participation at each stage Results (1st paragraph) |
|  |  | (c) Consider use of a flow diagram Figure 1 |
| Descriptive data | 14* | (a) Give characteristics of study participants (eg demographic, clinical, social) and information on exposures and potential confounders Table 1-2 |
|  |  | (b) Indicate number of participants with missing data for each variable of interest Table 1-2 |
|  |  | (c) Summarise follow-up time (eg, average and total amount) Not applicable |
| Outcome data | 15* | Report numbers of outcome events or summary measures over time Table 4 |
| Main results | 16 | (*a*) Give unadjusted estimates and, if applicable, confounder-adjusted estimates and their precision (eg, 95% confidence interval). Make clear which confounders were adjusted for and why they were included Figure 2 |
|  |  | (*b*) Report category boundaries when continuous variables were categorized  Footnote in Table 1-3 |
|  |  | (*c*) If relevant, consider translating estimates of relative risk into absolute risk for a meaningful time period Table 4 |
| Other analyses | 17 | Report other analyses done—eg analyses of subgroups and interactions, and sensitivity analyses Table 4 and Figure 2 |
| Discussion | | |
| Key results | 18 | Summarise key results with reference to study objectives Discussion (1^st^ paragraph) |
| Limitations | 19 | Discuss limitations of the study, taking into account sources of potential bias or imprecision. Discuss both direction and magnitude of any potential bias Discussion (7^th^ paragraph) |
| Interpretation | 20 | Give a cautious overall interpretation of results considering objectives, limitations, multiplicity of analyses, results from similar studies, and other relevant evidence Discussion (5-6 paragraph) |
| Generalisability | 21 | Discuss the generalisability (external validity) of the study results Discussion (7^th^ paragraph) |
| Other information | | |
| Funding | 22 | Give the source of funding and the role of the funders for the present study and, if applicable, for the original study on which the present article is based  Title page (Funding) |
