# Supplementary material for: Combining fecal immunochemical testing and questionnaire-based risk assessment in selecting participants for colonoscopy screening in the Chinese National Colorectal Cancer Screening Programs: A population-based cohort study
Source: PLoS Med. 2024 Feb 22;21(2):e1004340. doi: 10.1371/journal.pmed.1004340 (PMC10883529; doi:10.1371/journal.pmed.1004340)
Supplement: S1 Text — (DOCX) [file pmed.1004340.s004.docx]

# S1 Text: Quality control

Each institution documented participant information, including risk assessment, FIT and colonoscopy screening, and subsequent procedures. All data were transmitted to the coordinating center at China National Cancer Center through a web-based management system belonging to the National Cancer Prevention and Control Network (NCPCN) for double-checking and central reading. More than 3,000 hospitals across China were linked with NCPCN to double-check CRC information.
