# Supplementary material for: Combining fecal immunochemical testing and questionnaire-based risk assessment in selecting participants for colonoscopy screening in the Chinese National Colorectal Cancer Screening Programs: A population-based cohort study
Source: PLoS Med. 2024 Feb 22;21(2):e1004340. doi: 10.1371/journal.pmed.1004340 (PMC10883529; doi:10.1371/journal.pmed.1004340)
Supplement: S2 Text — (DOCX) [file pmed.1004340.s005.docx]

**S2 Text: Colorectal cancer risk score calculation**

The included risk factors, relative risks and exposure rates of risk factors were adjusted according to the characteristics of the Chinese population by the NCC expert group. Individuals with risk score over 1.5 and 4.0 in RF and RF-FIT strategies were defined as high risk score, respectively.

In RF strategy, each participant’s relative CRC risk, Harvard risk score, was calculated by dividing his or her individual cumulative risk score by the average risk score in males or females, which is defined as:

$$RR=\frac{RR_{l1}*RR_{l2}*...*RR_{\ln}}{\left[P_{1}*RR_{C1}+\left(1-P_{1}\right)\right]*\left[P_{2}*RR_{C2}+\left(1-P_{2}\right)\right]*...*\left[P_{n}*RR_{Cn}+\left(1-P_{n}\right)\right]}$$

where $RR_{\ln}$is the individual’s assigned relative risk for each risk factor (based on its presence or absence), $RR_{Cn}$corresponds to the consensus-based relative risk, and$Pn$represents the estimated prevalence of the risk factor in the general population (S1 Table).

In RF-FIT strategy, to enhance the efficiency of the questionnaire survey and mitigate potential recall bias in collecting diet, we streamlined the cancer risk score system, preserving only demographic variables and medical history, and incorporated the FIT test into the RF-FIT strategy. The risk score was calculated from S2 Table.
