# Supplementary material for: Combining fecal immunochemical testing and questionnaire-based risk assessment in selecting participants for colonoscopy screening in the Chinese National Colorectal Cancer Screening Programs: A population-based cohort study
Source: PLoS Med. 2024 Feb 22;21(2):e1004340. doi: 10.1371/journal.pmed.1004340 (PMC10883529; doi:10.1371/journal.pmed.1004340)
Supplement: S3 Text — (DOCX) [file pmed.1004340.s006.docx]

# S3 Text: Pathological examination

Abnormal findings were identified by pathological examination according to up-to-date clinical guidelines. The pathological examination confirmed all abnormal findings discovered after colonoscopy following up-to-date clinical guidelines. Pathologists were required to complete the highly standardized forms to collect pathology results. For undiscernible difficult cases, consultation by the National Cancer Center of China expert panel was conducted, and review reports were transferred to the respective physicians about the consultation results. This study defines advanced adenomas as at least one adenoma >10 mm or one with villous components or high-grade dysplasia. In addition, we categorized advanced neoplasms (CRC and advanced adenomas) were categorized into the proximal colon and distal colon locations. The largest one was used to categorize the location if multiple similarly advanced neoplasms lesions were present.
